# Are Micro-Organisms Scavengers, or the Producers of Pathological Conditions?

**Published:** 1886-08

**Authors:** 


### ARE MICRO-ORGANISMS SCAVENGERS, OR THE PRODUCERS OF PATHOLOGICAL CONDITIONS?

In the new dictionary of Practical Surgery, by Christopher Heath, under the head of "Abscess," we find the following:

"It must be remarked that micro-organisms are always present in these cases (septic abscesses), and that we are totally unable to say how far and in what way they may be irritating in themselves, independently of the acid fluids which accompany their growth."

It would seem as though this were a late day to begin to raise questions concerning the nature and origin of the acids which accompany the proliferation of micro-organisms. Miller and Black have demonstrated that they are the *products* of the organisms themselves, and the former has analyzed some of them and determined their character. Further, he has produced certain oral and gastric disturbances by inoculation with cultures of the organisms. It has been urged in the past that it was impossible to say whether or not the organisms attending certain pathological conditions were cleansing in their nature, and their office the removal of the septic products of disease. It was loudly asserted that their habitat was disordered tissue, and that they could only proliferate in pathological conditions.

There was much of probability in the theory, but it has been absolutely demonstrated as false in certain conditions, and so far as we know, Miller was the first to do this. He proved that veritable decay and destruction of hard tissues could be brought about by infection with pure cultures of the organisms, and at the same time he demonstrated the exact nature and origin of the fluids in which they existed, and the acids which accompanied them. The organisms themselves having been shown to produce the acids, there was no longer any question about their pathogenic character.

It has always been our boast that general medicine was indebted to dentistry, not only for anæsthesia, but for the demonstration of other medical principles, and the first absolute chemical and physiological demonstration of the true character of the disease germs of the human system is another mark to our credit, and a matter in which every dentist should feel a just pride.

